# Supplementary material for: Rac1/ROCK-driven membrane dynamics promote natural killer cell cytotoxicity via granzyme-induced necroptosis
Source: BMC Biol. 2021 Jul 30;19:140. doi: 10.1186/s12915-021-01068-3 (PMC8323222; doi:10.1186/s12915-021-01068-3)
Supplement: Supplementary file 1 — Additional file 1: Figure S1. FACS analysis of NK cell-induced target cell death. Figure S2- Effect of MICB knockdown on NK cell killing. Figure S3. Cancer target-dependent kinetics and phenotypes of NK cell killing. Figure S4. NK cell killing under NK CD16 neutralization. Figure S5. Membrane distributions of HLA-A,B,C in MCF7 cells and KIR2D in primary NK cells by immunostaining. Figure S6. Additional representative immunofluorescence images of phospho-MLKL. Figure S7. Full western blots. [file 12915_2021_1068_MOESM1_ESM.pdf]

Supplementary Information for

Rac1/ROCK-driven membrane dynamics promote Natural Killer cell cytotoxicity via granzyme-induced necroptosis

Yanting Zhu[1], Jun Xie[1] and Jue Shi[1*]

[1] Center for Quantitative Systems Biology, Department of Physics and Department of Biology, Hong Kong Baptist University, Hong Kong, China

* Corresponding author: Jue Shi, jshi@hkbu.edu.hk

This supplementary file includes supplementary Figures S1-S7.

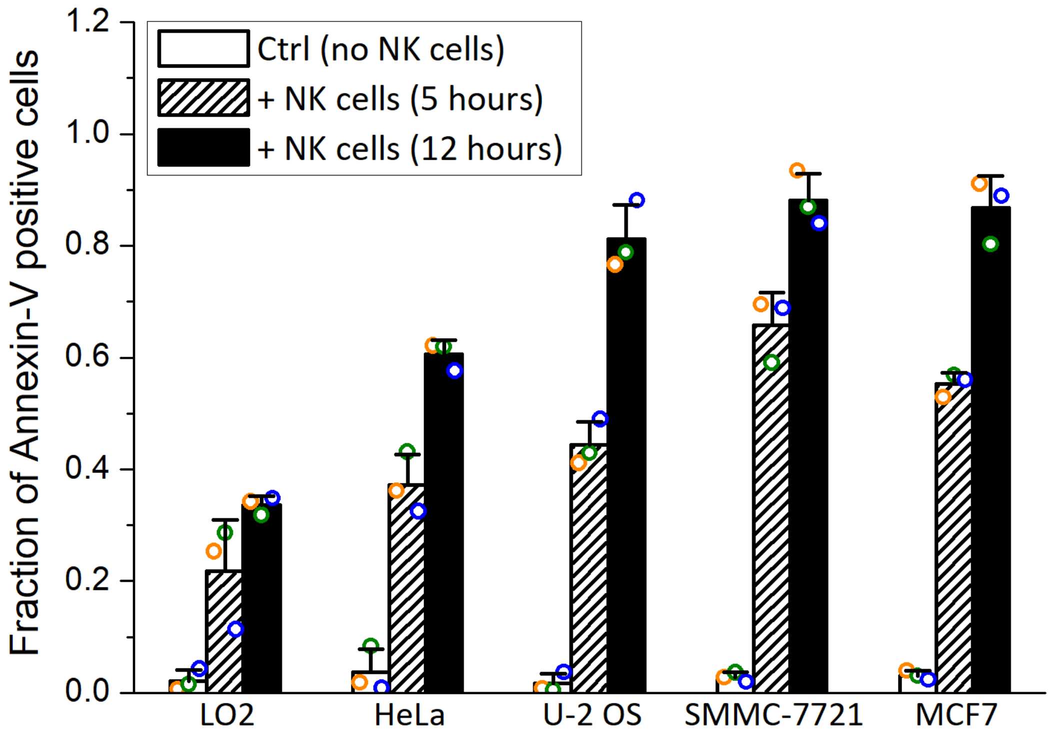

Supplementary Figure S1. Flow cytometry analysis of total target cell death in response to primary NK cells by Annexin V staining. Cells were harvested after the indicated durations (i.e., 5 hours and 12 hours) of co-culture with primary NK cells and then stained with Annexin V, which marks both apoptotic and necrotic cells. > 10^4 cells were counted by FACS analysis for each condition and data were averaged from 3 independent experiments and each experiment was conducted with NK cells from a different healthy donor (denoted by different color symbols). This FACS data confirmed the variable target cell death in response to NK cell killing that we observed by single cell imaging. Moreover, target cells under control condition, i.e., without NK cells, in general showed less than 5% spontaneous cell death, indicating good target cell health and that the spontaneous cell death did not significantly affect the NK cell-induced cell death that we analyzed in this study.

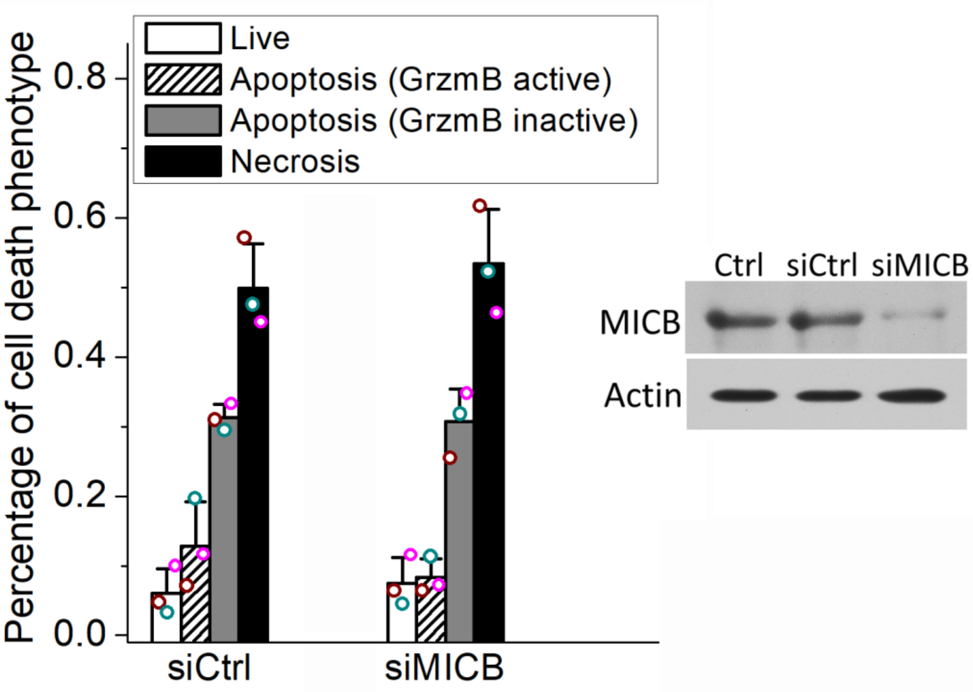

Supplementary Figure S2. MCF7 cells with or without MICB knockdown were co-cultured with primary NK cells for 12 hours and the resulting MCF7 cell killing was compared. The data showed that MICB knockdown did not attenuate the extent of necrotic death of MCF7 cells induced by primary NK cells. Data were averaged from 3 independent experiments with NK cells from 3 different donors (denoted by different color symbols). MICB was knocked down by RNAi and the knockdown efficiency is larger than 95%, as shown in the western blot.

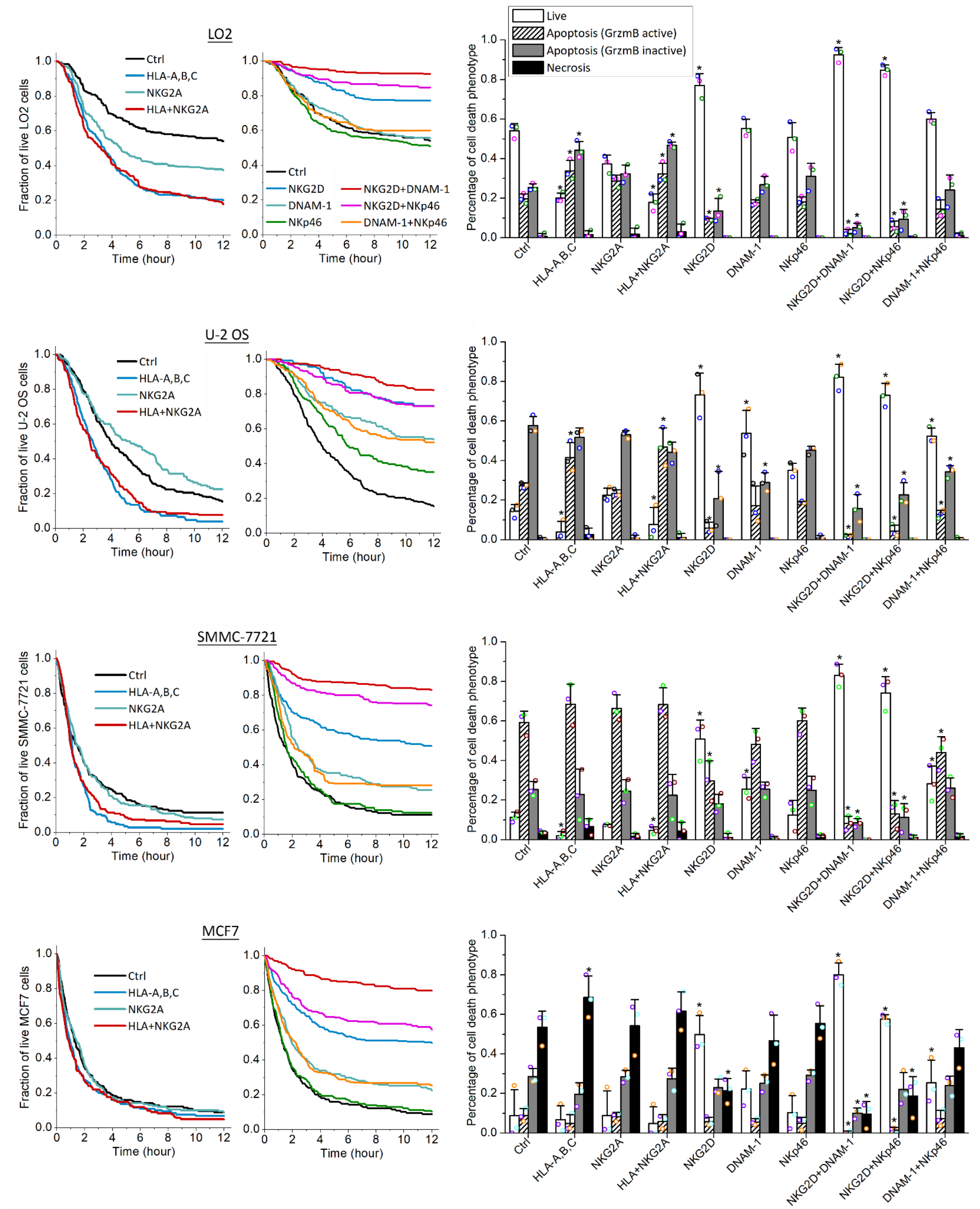
LO2
U-2 OS
SMMC-7721
MCF7
Fraction of live LO2 cells
Fraction of live U-2 OS cells
Fraction of live SMMC-7721 cells
Fraction of live MCF7 cells
Time (hour)
Ctrl
HLA-A,B,C
NKG2A
HLA+NKG2A
NKG2D
DNAM-1
NKp46
NKG2D+DNAM-1
NKG2D+NKp46
DNAM-1+NKp46
Percentage of cell death phenotype
Live
Apoptosis (GrzmB active)
Apoptosis (GrzmB inactive)
Necrosis

Supplementary Figure S3. Cumulative survival curves of LO2, U-2 OS, SMMC-7721 and MCF7 in co-culture with primary NK cells in the presence of single or double blockage of the inhibitory receptors (left panel) or activating receptors (middle panel). The activating receptor blocking conditions for all target cell lines were similarly color coded as shown under LO2. Right panel: Distributions of the live and dead target cells killed by the three distinct cytotoxic modes after 12 hours of co-culture with NK cells under the indicated treatment conditions. Data plotted were averaged from 3 independent imaging experiments and the error bars are standard deviations. Symbols with the same colors denote data acquired with NK cells from the same healthy donors. P value was obtained by the student's t test comparing the neutralizing antibody treatment condition with control. * $P < 0.001$

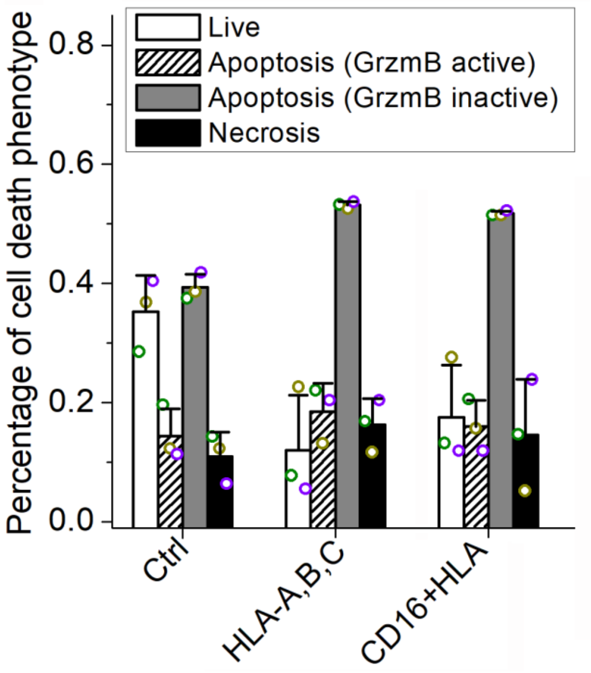

Supplementary Figure S4. In order to examine whether the neutralizing antibody treatment enhances target cell death by NK cell-mediated Antibody-Dependent Cell-mediated Cytotoxicity (ADCC), we treated NK cells with CD16 neutralizing antibody, as CD16 is the key Fcγ receptor that mediates ADCC by NK cells. Data shown in the above figure were averaged from 3 independent imaging experiments with NK cells purified from 3 different healthy donors (denoted by symbols of different colors). Blocking CD16 did not significantly attenuate the enhanced target cell death observed under HLA-A,B,C neutralization, e.g., in HeLa cells (the cancer cell line that showed the highest increase in cell death under HLA-A,B,C neutralization). Therefore, our data suggest that ADCC did not significantly contribute to the enhanced target cell death that we observed under the neutralizing antibody treatment.

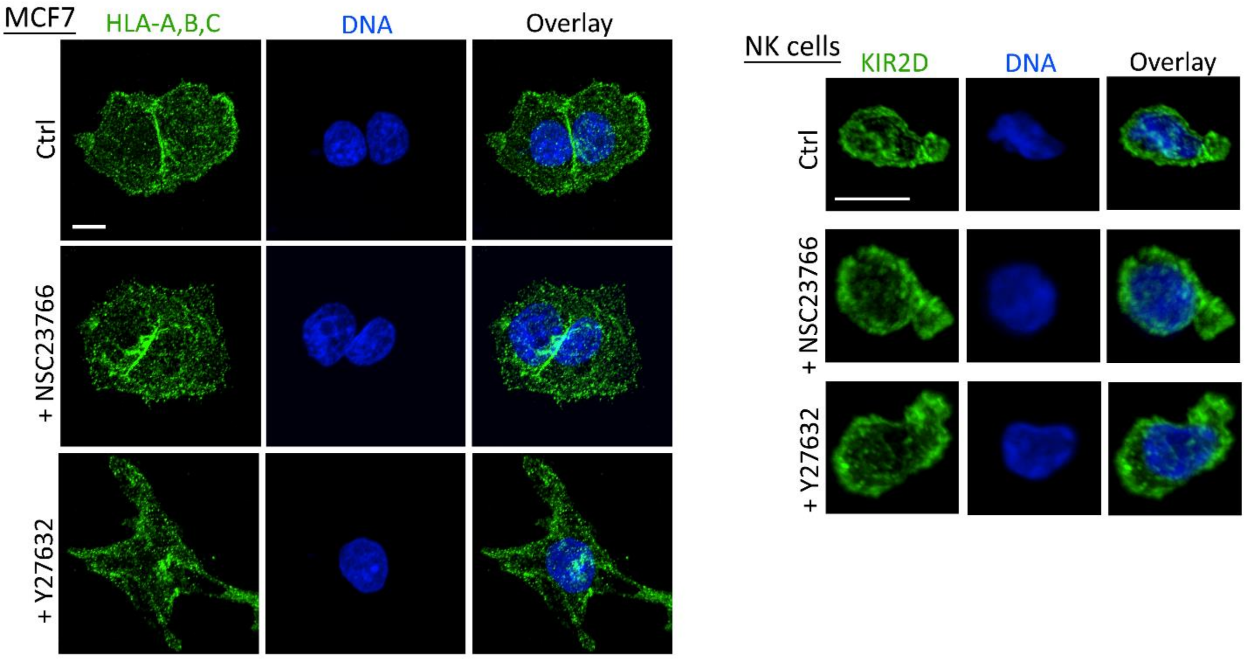

Supplementary Figure S5. Membrane distributions of HLA-A,B,C in MCF7 cells and KIR2D in primary NK cells were analyzed by immunostaining. MCF7 cells and NK cells under control and the two inhibitor treatment conditions were stained with the respective antibodies and imaged by confocal microscopy. The white scale bars are 10 μm. Under the treatment of the Rac1 inhibitor, NSC23766, and the ROCK inhibitor, Y27632, patterns of HLA-A,B,C membrane distribution in MCF7 cells and KIR2D distribution in primary NK cells were largely similar to those under the control condition, indicating these two inhibitors did not significantly alter the membrane distributions of NK ligands and receptors.

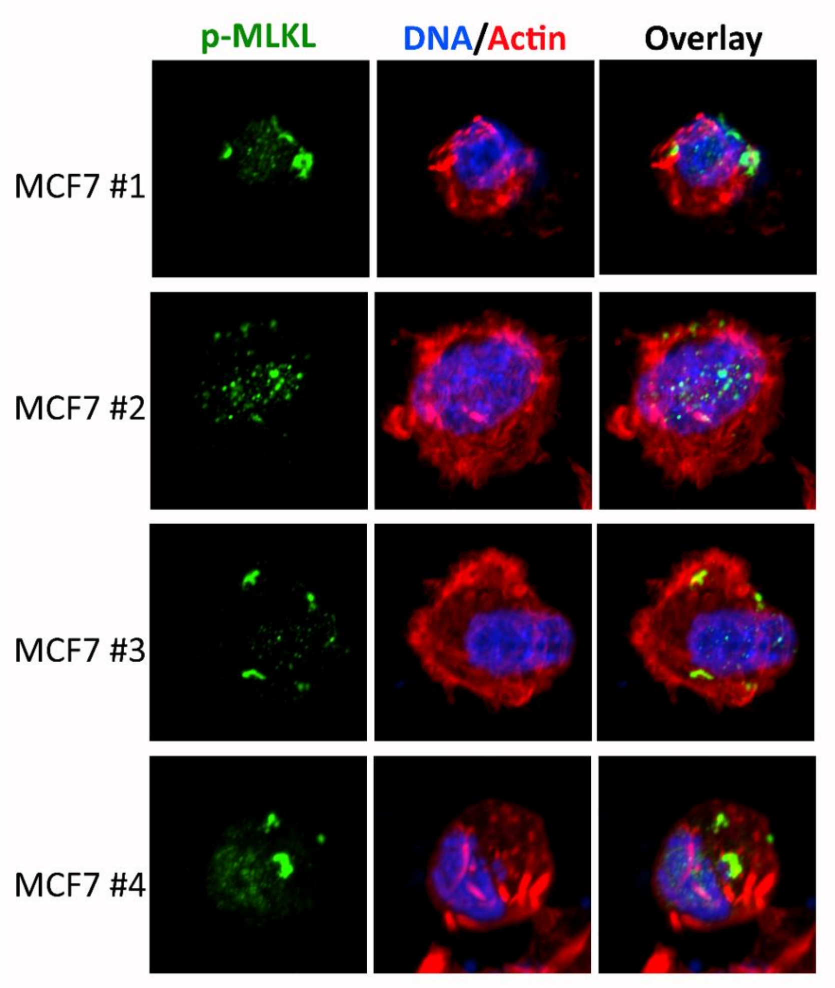

Supplementary Figure S6. Additional representative immunofluorescence images of MCF7 cells in co-culture with primary NK cells. p-MLKL puncta (shown in green) were observed in dying MCF7 cells upon NK cell treatment.

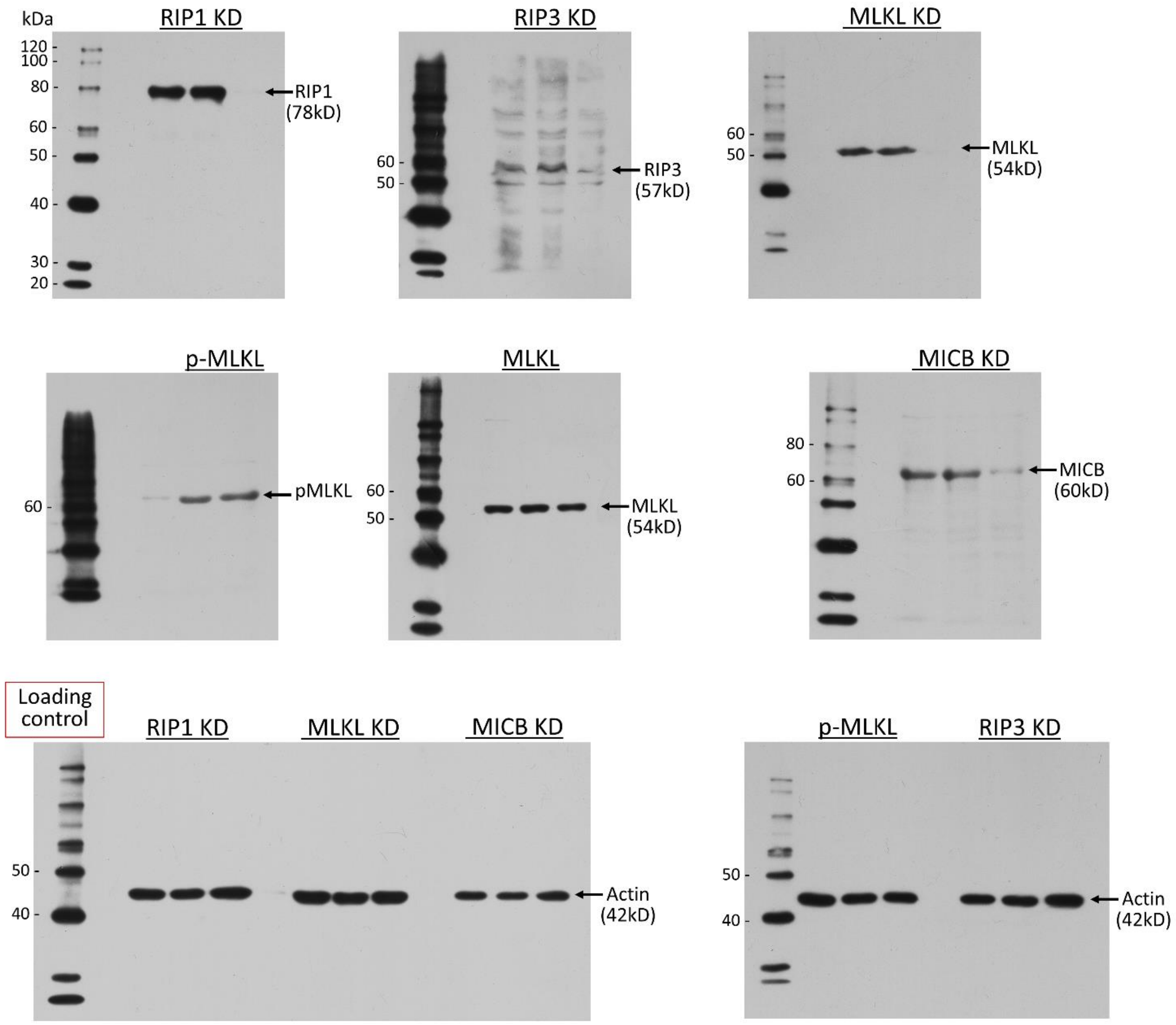

Supplementary Figure S7. Full western blots with molecular weight markers for data shown in the main text (Fig. 5C and 5D) and supplementary materials (supplementary Figure S2). The corresponding molecular weights of the full marker (MagicMark XP Western Protein Standard, Thermo Fisher) are indicated in the western blot on the top left.
